# Cuvier

**Published:** 1844-09-07

**Authors:** 


CUVIER.

Frequently *absent*, as we say, in the midst of his family, its members always made war upon the philosopher on these occasions. It happened, particularly that he showed himself more than usually absorbed in his own reflections at the period when he was engaged in assigning to precise species the fragments of the fossil bones discovered in the quarries of Montmartre. He once, in particular, spent several days searching after the fore foot of a certain skeleton of which he had already collected numerous fragments, when he fell into one of his absent moods, Madlle. Duvancel would rally him, and giving him a gentle salute, would say, "Well, and art thou still seeking after that fore foot of thine?"—*Bourdon, Illustres Médecins.*

A public funeral was given at Manchester, on Monday last, to the remains of the late Dr. Dalton. There were no less than a hundred private carriages, inclnding that of the Corporation, and the various societies in town formed part of the procession to the Ardwich Cemetery, where his remains were interred. The shops and warehouses in the line of road to the place of interment were closed, manifesting a marked desire to pay every respect to the philosopher's memory.—*Lon. Med. Times.*

